# The neutrophil percentage-to-albumin ratio predicts mortality in US adults with rheumatoid arthritis: Results from NHANES 2003–2018

**DOI:** 10.1097/MD.0000000000049453

**Published:** 2026-07-17

**Authors:** Beiqi Xu, Jun Dou, Yufan Wu, Yanzhi Deng, Lilan Wang, Ximei Wang, Guang Yang, Dan Xie

**Affiliations:** a Department of Emergency Medicine, Kunshan Hospital Affiliated to Nanjing University of Chinese Medicine, Kunshan, Jiangsu, China; b Department of Rheumatology and Immunology, Children’s Hospital of Soochow University, Suzhou, Jiangsu, China; c Department of Rheumatology and Immunology, Changzhou Affiliated Hospital of Nanjing University of Chinese Medicine, Changzhou, Jiangsu, China; d Department of Traditional Chinese Medicine, The First People’s Hospital of Huzhou, Huzhou, Zhejiang, China.

**Keywords:** albumin, inflammation, mortality, Neutrophil, rheumatoid arthritis

## Abstract

The neutrophil percentage-to-albumin ratio (NPAR), calculated from neutrophil percentage and serum albumin levels, has been proposed as an inflammation-related indicator. Despite increasing interest in NPAR as an inflammatory marker, its prognostic relevance in rheumatoid arthritis (RA) remains insufficiently understood. The objective of this study was to investigate the prognostic relevance of NPAR in US adults with RA, particularly regarding all-cause and cardiovascular mortality, and to evaluate the prognostic performance of both NPAR and the neutrophil-to-lymphocyte ratio. Data were obtained from the National Health and Nutrition Examination Survey (NHANES) 2003–2018. RA status was identified according to participants’ self-reported physician diagnosis, consistent with previous NHANES investigations. NPAR was calculated as neutrophil percentage × 100/albumin concentration (g/dL). Statistical analyses incorporated the NHANES complex survey framework, including weighting, stratification, and clustering procedures. Missing covariate data were addressed using multiple imputation. Associations between NPAR and mortality outcomes were examined using survey-weighted Cox regression models and restricted cubic spline analyses. The analysis included 2060 adults with RA, among whom 484 died during follow-up and 1576 remained alive. All-cause and cardiovascular mortality risks increased across NPAR levels and remained highest in the top quartile after multivariable adjustment. Kaplan–Meier analysis demonstrated significantly reduced survival probabilities among participants in the highest NPAR quartile, except for those with diabetes. NPAR exhibited a positive linear association with risks of all-cause and cardiovascular death in the restricted cubic spline models. Calculated AUCs reached 0.63 for the former and 0.66 for the latter, suggesting modest discriminatory power akin to neutrophil-to-lymphocyte ratio. NPAR was independently linked to mortality during follow-up in US patients with RA. As a routinely available biomarker, it may have prognostic relevance, although its predictive ability was relatively limited.

## 1. Introduction

Rheumatoid arthritis (RA) is a chronic inflammatory autoimmune disorder that mainly affects synovial joints and can ultimately result in pain, joint deformity, and loss of functional capacity.^[1]^ The worldwide burden of RA continues to increase, with an estimated 17.6 million cases reported in 2020, marking a 14.1% increase from 1990. RA continues to impose an increasing global health burden, and the number of people affected worldwide is projected to rise to 31.7 million by 2050.^[2]^ RA is associated with shortened survival, with cardiovascular disease (CVD) accounting for a substantial proportion of deaths among affected individuals.^[3-5]^ Identifying factors correlated to the development and prognosis of RA is essential for improving disease management and therapeutic strategies. Persistent systemic inflammation is a hallmark of RA pathogenesis and progression and has also been linked to increased cardiovascular risk and mortality.^[6-8]^ Accordingly, inflammatory biomarkers may provide prognostic value for patients with RA.

The neutrophil percentage-to-albumin ratio (NPAR) is a biomarker of systemic inflammation and the responses of the innate (neutrophils) and adaptive (lymphocytes) immune systems.^[9]^ NPAR has been linked to poor prognosis in a variety of diseases.^[10,11]^ The neutrophil-to-lymphocyte ratio (NLR), an index derived from circulating neutrophil and lymphocyte counts, is widely considered to reflect systemic inflammation and immune dysregulation and has been linked to poor prognosis.^[12]^ In RA, NLR is positively correlated with disease activity markers such as C-reactive protein and erythrocyte sedimentation rate, which are widely used to reflect nonspecific systemic inflammation and are incorporated into disease activity scores. NLR serves as a surrogate marker for systemic inflammation and immune dysfunction by quantifying the balance between neutrophils and lymphocytes.^[13,14]^ However, the role of NPAR in predicting mortality risk in RA remains unclear. In addition, its prognostic performance compared with NLR has not been directly assessed.

Given the need for readily obtainable and clinically practical prognostic indicators for RA, this study used data from the National Health and Nutrition Examination Survey (NHANES) 2003–2018 to examine the relationship of NPAR with all-cause and cardiovascular mortality among US adults with RA and the prognostic performance of NPAR and NLR in this population.

## 2. Materials and methods

### 2.1. Study design and population

The National Health and Nutrition Examination Survey is a continuous program conducted by the National Center for Health Statistics under the Centers for Disease Control and Prevention. It collects data through interviews, physical examinations, laboratory testing, and questionnaires, providing nationally representative information on the health and nutritional status of the US civilian population for public health monitoring and policy development. Data and documentation are available at http://www.cdc.gov/nchs/nhanes.htm.

The study protocol received approval from the Institutional Review Board of the Centers for Disease Control and Prevention, and written informed consent was obtained from all participants prior to participation. The study was reviewed by the IRB of our institution. The study was exempt from full IRB review.

**Figure 1.**
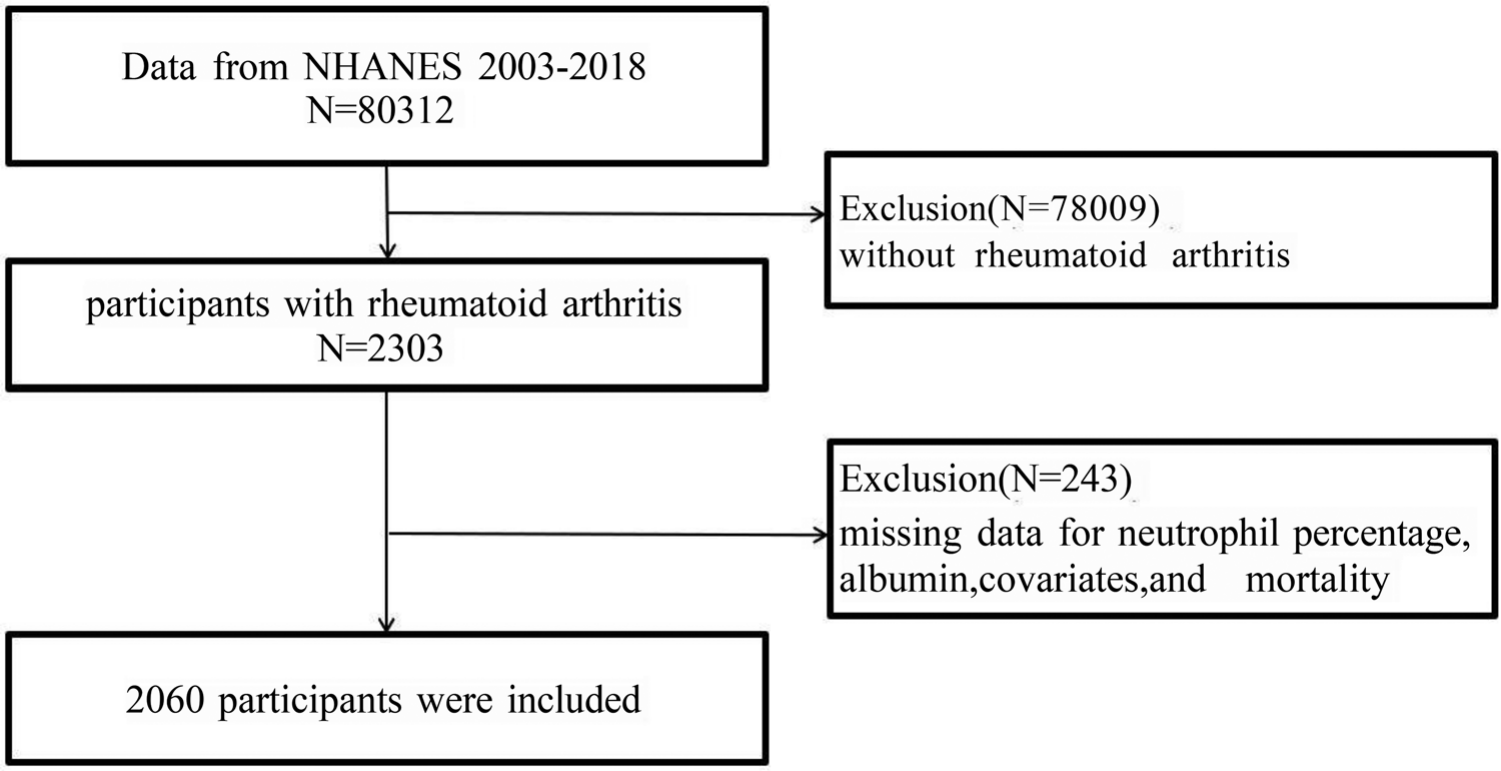
Flowchart of patient selection. NHANES = National Health and Nutrition Examination Survey.

Participants were excluded if they were younger than 20 years or lacked data on neutrophil percentage, albumin, covariates, and mortality. The flowchart of patient selection is depicted in Figure 1.

### 2.2. Definition of main variables

The participants who answered “yes” to the following question were defined as having RA: “Has a doctor or other health professional ever told you that you have arthritis?” NPAR was calculated using the formula: neutrophil percentage × 100/albumin concentration (g/dL).

The primary endpoint was cardiovascular mortality. Mortality information was obtained through the publicly available National Death Index-linked mortality files (https://www.cdc.gov/nchs/data-linkage/mortality-public.htm). Follow-up time was defined as the interval between the NHANES examination date and either the date of death or December 31, 2019, corresponding to the latest National Death Index update.

The participants who answered “yes” to 2 of the following questions were defined as having heart disease: “Have you ever been diagnosed with congestive heart failure?,” “Have you ever been diagnosed with coronary heart disease?,” “Have you ever experienced angina/angina pectoris?,” and “Have you ever had a heart attack?”

### 2.3. Assessment of covariates

Demographic factors included sex, age, and race/ethnicity (Mexican American, other Hispanic, non-Hispanic White, non-Hispanic Black, and other races). Socioeconomic factors included educational attainment, marital status, and poverty–income ratio (PIR), categorized as low (<1.30), middle (1.30–3.49), and high (≥3.50). Lifestyle and health-related factors included body mass index (BMI), CVD, type 2 diabetes mellitus (T2DM), hypertension, smoking status, and NPAR. Hypertension was defined as an average systolic blood pressure of ≥140 mm Hg, an average diastolic blood pressure of ≥90 mm Hg, or the current use of antihypertensive medications. Missing covariate data were handled using multiple imputation by chained equations (5 imputations).

### 2.4. Cox proportional hazards models

Three Cox proportional hazards models were constructed to examine the associations of NPAR with all-cause and cardiovascular mortality after controlling for potential confounders. The first model was unadjusted. Model 1 was adjusted for age, sex, and ethnicity. Model 2 was adjusted for age, sex, ethnicity, education level, marital status, PIR, BMI, alcohol consumption, smoking, hypertension, heart disease, and diabetes.

### 2.5. Statistical analysis

Continuous variables were presented as mean ± standard deviation, and categorical variables were presented as numbers and percentages. Differences between groups were compared using the chi-square test or *t*-test as appropriate. Survival probabilities by NPAR quartile were estimated using Kaplan–Meier curves. Subgroup analyses were conducted to explore potential effect modification by covariates. The relationships of NPAR with mortality outcomes were further illustrated using restricted cubic spline curves. All analyses were performed using R Statistical Software version 4.2.2 (http://www.R-project.org, The R Foundation) and Free Statistics software version 2.0 (Beijing, China). A 2-sided *P*-value of <.05 was considered statistically significant.

## 3. Results

### 3.1. Study population

Baseline characteristics of participants, grouped by survival status (alive or deceased), are summarized in Table 1. The cohort included 2060 individuals (1576 alive and 484 dead; 41.8% male and 58.2% female). Before multiple imputation, 243 participants (10.5%) were excluded due to missing covariate data, as shown in Figure 1. All variables, except for sex, BMI, T2DM, smoking status, and alcohol consumption, showed a significant difference between living and deceased patients. The patients who died were more likely to be aged 60 years or older, married, non-Hispanic White, and to have a college or university degree. Additionally, the patients who died exhibited a higher incidence of heart disease and NPAR. Hypertension was not a risk factor for death (all *P* < .01).

**Table 1:** Characteristics of the study participants.

Variable	Total N = 2060	Alive N = 1576	Death N = 484	*P*
Sex, n (%)				.122
Male	862 (41.8)	625 (40.9)	237 (45.5)	
Female	1198 (58.2)	951 (59.1)	247 (54.5)	
Age, yr, n (%)				<.001
20–59	832 (53.2)	762 (61.3)	70 (21.2)	
≥60	1228 (46.8)	814 (38.7)	414 (78.8)	
Ethnicity, n (%)				<.001
Mexican American	318 (7.2)	270 (8.0)	48 (4.3)	
Other Hispanic	181 (4.7)	159 (5.3)	22 (2.4)	
Non-Hispanic White	845 (66.1)	571 (63.6)	274 (76.0)	
Non-Hispanic Black	594 (16.3)	466 (16.7)	128 (14.6)	
Other Race	122 (5.7)	110 (6.5)	12 (2.7)	
Education level, n (%)				<.001
<High school	704 (23.9)	494 (21.3)	210 (34.1)	
≥High school	1356 (76.1)	1082 (78.7)	274 (65.9)	
Married, n (%)	1009 (45.5)	817 (42.1)	192 (58.7)	<.001
Poverty–income ratio, n (%)				<.001
<1.3	931 (35.1)	709 (34.1)	222 (39.0)	
1.3–3.5	715 (35.5)	521 (33.7)	194 (42.9)	
>3.5	414 (29.4)	346 (32.3)	68 (18.1)	
Body mass index, kg/m^2^, n (%)				.016
<25	445 (23.3)	309 (22.4)	136 (27.1)	
25–30	636 (30.5)	474 (29.3)	162 (35.5)	
≥30	979 (46.1)	793 (48.4)	186 (37.4)	
Heart disease, n (%)	430 (19.6)	259 (14.8)	171 (38.1)	<.001
Diabetes, n (%)	696 (61.6)	555 (59.7)	141 (69.2)	.008
Hypertension, n (%)	1529 (68.6)	1122 (64.8)	407 (83.7)	<.001
Smoking, n (%)	1131 (58.1)	836 (56.7)	295 (63.6)	.026
Alcohol consumption, n (%)	882 (48.7)	712 (52.0)	170 (35.4)	<.001
NPAR	14.5 ± 2.9	14.2 ± 2.7	15.5 ± 3.2	<.001

Data are expressed as means ± standard deviations or unweighted frequency counts (weighted percentages) as appropriate. The groups were compared using an independent *t*-test for continuous variables and the Rao–Scott chi-square test for categorical variables.

NPAR = neutrophil percentage-to-albumin ratio, PIR = poverty–income ratio.

### 3.2. Association between NPAR and mortality

The results showed that the risk of all-cause mortality was significantly higher in the NPAR group in the highest quartile than in the Q1 group (hazard ratio [HR] = 2.75, 95% confidence interval [CI] = 1.96–3.85, *P* < .001) even after adjusting for age, sex, and race (HR = 2.39, 95% CI = 1.68–3.40, *P* < .001) and adjusting for the variables in model 1 plus education level, PIR, and BMI (HR = 2.25, 95% CI = 1.57–3.23, *P* < .001).

**Figure 2.**
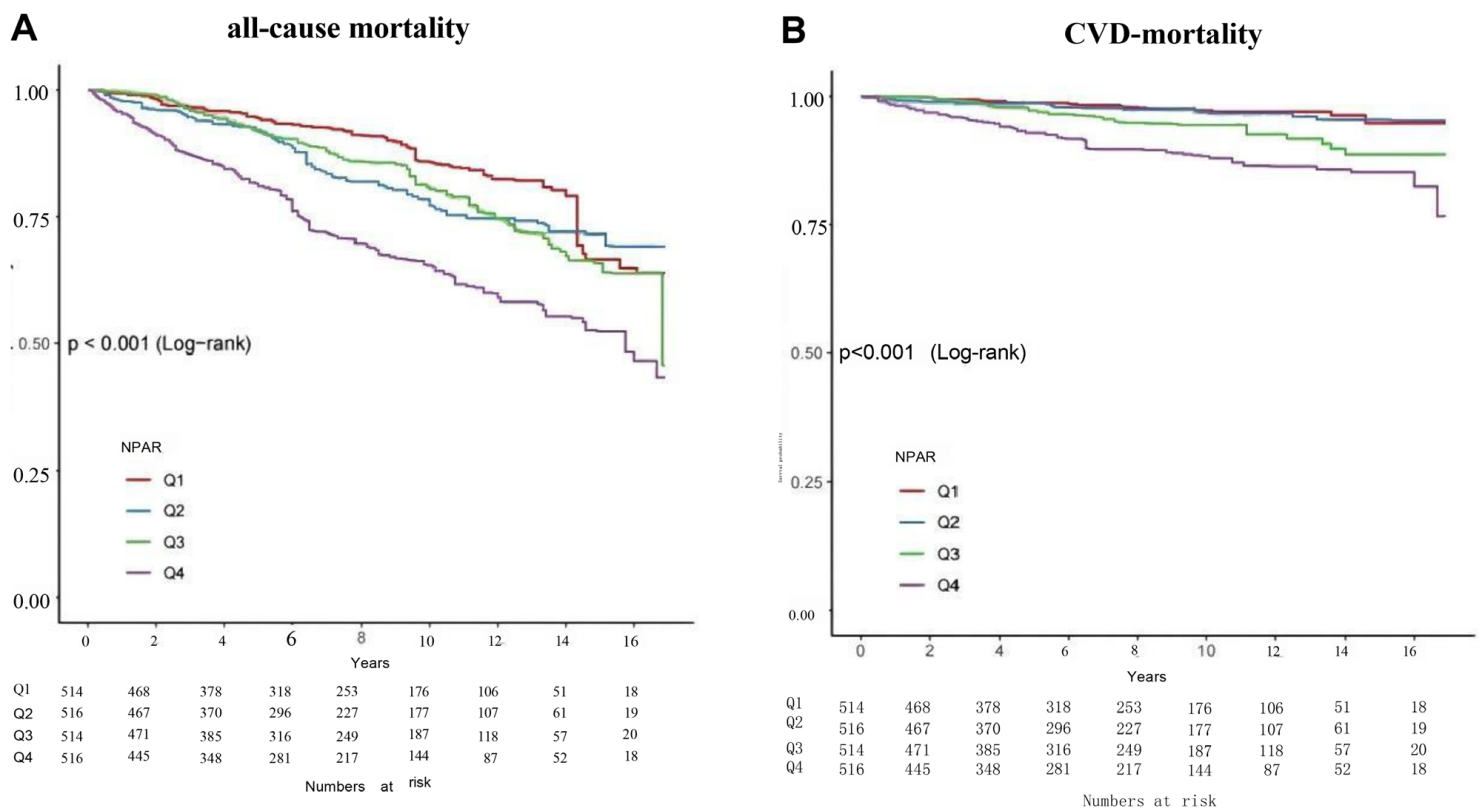
(A, B) Kaplan–Meier curves of all-cause and cardiovascular mortality in patients with rheumatoid arthritis. CVD = cardiovascular disease.

The risk of cardiovascular mortality was significantly higher in the Q3 group (HR = 2.23, 95% CI = 1.14–4.98, *P* = .021) and the Q4 group (HR = 4.84, 95% CI = 2.52–9.29, *P* < .001) than in the Q1 group, even after adjusting for all covariates (*P* < .001), with the Q4 group having the highest risk of cardiovascular mortality (Table 2). Kaplan–Meier analysis revealed that both all-cause and cardiovascular mortality were highest in the Q4 group compared with the other quartiles (*P* < .001; Fig. 2).

**Table 2:** Association between NPAR and mortality.

		Unadjusted		Model 1		Model 2	
		HR (95% CI)	*P*	HR (95% CI)	*P*	HR (95% CI)	*P*
All-cause mortality							
NPAR	continuous	1.14 (1.10, 1.19)	<.001	1.15 (1.10, 1.19)	<.001	1.14 (1.09, 1.19)	<.001
	Q1	Ref		Ref		Ref	
	Q2	1.39 (0.95, 2.03)	.094	1.26 (0.88, 1.79)	.206	1.31 (0.94, 1.83)	.110
	Q3	1.38 (0.99, 1.91)	.056	1.36 (0.98, 1.89)	.062	1.37 (0.96, 1.95)	.081
	Q4	2.75 (1.96, 3.85)	<.001	2.39 (1.68, 3.40)	<.001	2.25 (1.57, 3.23)	<.001
	*P* for trend		<.001		<.001		<.001
CVD mortality							
NPAR	continuous	1.22 (1.15, 1.31)	<.001	1.23 (1.15, 1.32)	<.001	1.23 (1.14, 1.32)	<.001
	Q1	Ref		Ref		Ref	
	Q2	1.20 (0.57, 2.50)	.634	1.09 (0.52, 2.28)	.822	1.09 (0.50, 2.34)	.835
	Q3	2.39 (1.14, 4.98)	.021	2.42 (1.15, 5.10)	.020	2.56 (1.17, 5.62)	.019
	Q4	4.84 (2.52, 9.29)	<.001	4.17 (2.21, 8.21)	<.001	3.59 (1.81, 7.11)	<.001
	*P* for trend		<.001		<.001		<.001

Model 1 was adjusted for age, sex, and race/ethnicity. Model 2 was adjusted for the variables in model 1 as well as education level, marital status, PIR, BMI, alcohol consumption, cigarette smoking, hypertension, CVD, and type 2 diabetes mellitus.

BMI = body mass index, CI = confidence interval, CVD = cardiovascular disease, HR = hazard ratio, NPAR = neutrophil percentage-to-albumin ratio, PIR = poverty–income ratio.

### 3.3. Subgroup analyses

**Figure 3.**
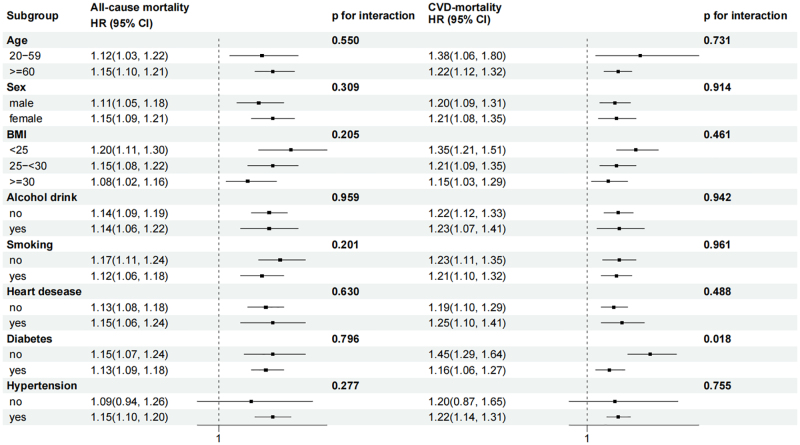
Subgroup analysis of all-cause and cardiovascular mortality in patients with rheumatoid arthritis.

In subgroup analyses stratified by age, sex, BMI, alcohol drinking, smoking status, CVD, T2DM, and hypertension, NPAR was significantly positively associated with the risk of all-cause and cardiovascular mortality across all subgroups (Fig. 3). As shown in Figure 3, interaction tests revealed no significant effect modification by age (*P* for interaction = .550), sex (*P* = .309), BMI (*P* = .205), alcohol consumption (*P* = .959), CVD (*P* = .630), or hypertension (*P* = .277). However, a significant interaction was observed between NPAR and T2DM for cardiovascular mortality (*P* for interaction = .018), suggesting that the association may be stronger in individuals with T2DM. Taken together, the subgroup analyses showed generally consistent patterns.

### 3.4. Nonlinear relationship between NPAR and mortality

**Figure 4.**
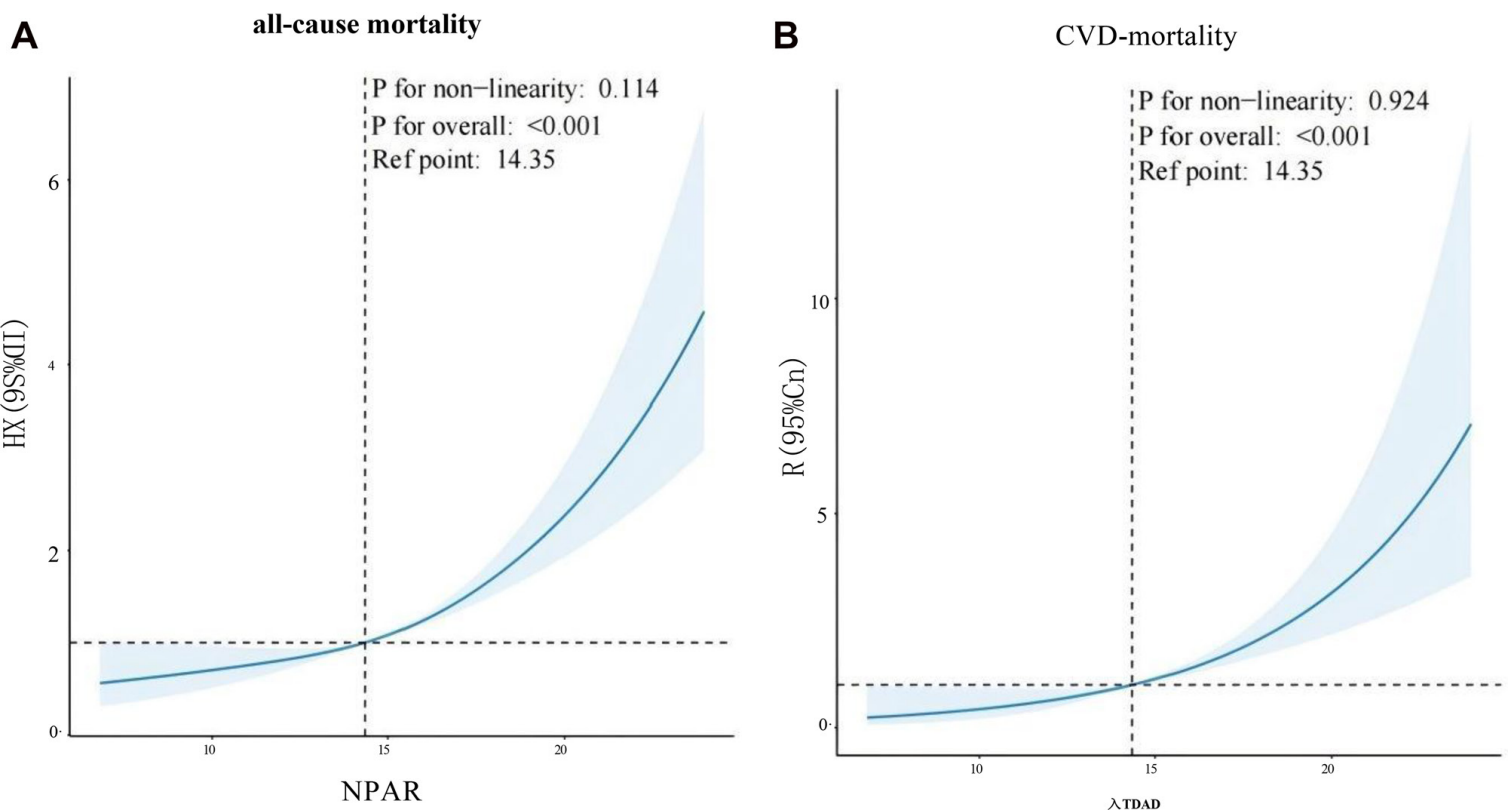
(A, B) Restricted cubic spline curves showing the association between the neutrophil percentage-to-albumin ratio and all-cause and cardiovascular mortality in patients with rheumatoid arthritis. CVD = cardiovascular disease, NPAR = neutrophil percentage-to-albumin ratio.

Restricted cubic spline analyses demonstrated a significant linear association between NPAR and all-cause mortality in the overall cohort (*P* < .001), with no evidence of nonlinearity (*P* for nonlinearity = .114; Fig. 4A). A similar linear relationship was observed between NPAR and cardiovascular mortality (*P* < .001), also without evidence of nonlinearity (Fig. 4B). The prognostic performance of this inflammatory biomarker differed between all-cause and cardiovascular mortality in individuals with RA.

### 3.5. Predictive value of NPAR and NLR

**Figure 5.**
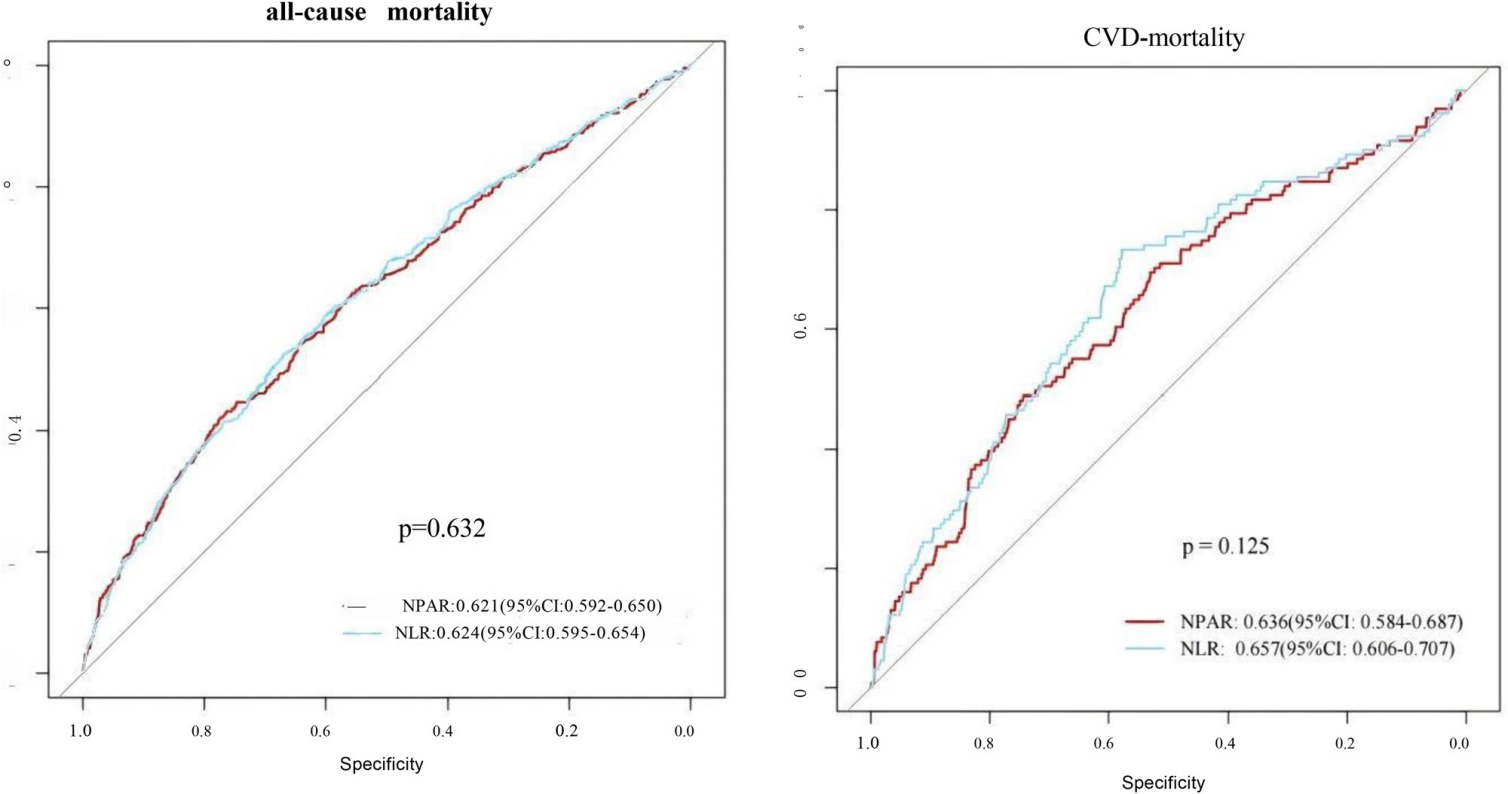
Receiver operating characteristic curve analysis of the ability of the neutrophil percentage-to-albumin ratio and the neutrophil-to-lymphocyte ratio to predict all-cause and cardiovascular mortality in patients with rheumatoid arthritis. CI = confidence interval, NLR = neutrophil-to-lymphocyte ratio, NPAR = neutrophil percentage-to-albumin ratio.

Receiver operating characteristic curves showed modest predictive discrimination for NPAR (AUC for all-cause mortality: 0.63; AUC for cardiovascular mortality: 0.66). NLR showed similar performance (AUC for all-cause mortality: 0.62; AUC for cardiovascular mortality: 0.64). There were no statistically significant differences between NPAR and NLR in predicting all-cause mortality (*P* = .632) or cardiovascular mortality (*P* = .125; Fig. 5). These AUC values indicate that both NPAR and NLR have limited ability to discriminate between patients who will die and those who will survive, and thus should be interpreted cautiously as prognostic markers.

## 4. Discussion

The analysis of nationally representative data from NHANES 2003–2018 demonstrated that elevated NPAR is an independent predictor of all-cause and cardiovascular mortality in US adults with RA. A clear dose–response pattern was observed, with participants in the highest NPAR quartile (Q4) showing a 2.75-fold higher risk of all-cause mortality (95% CI: 1.96–3.85) and a 4.84-fold higher risk of cardiovascular mortality (95% CI: 2.52–9.29). This association persisted after adjustment for demographic, clinical, and lifestyle covariates. However, the predictive discrimination of NPAR was modest, with AUC values below 0.70, suggesting limited clinical utility as a standalone prognostic tool. These results underscore the potential utility of NPAR as a prognostic biomarker for RA.

Increasing evidence suggests that both innate and adaptive immune responses contribute to the development of autoimmune diseases, and NPAR may reflect the underlying inflammatory milieu.^[15,16]^ Chronic systemic inflammation and dysregulated innate immunity play key roles in the pathogenesis of RA.^[17]^

Neutrophils are early responders that rapidly accumulate at sites of injury or infection. In RA, they infiltrate the synovial joints and become activated, thereby promoting persistent inflammation and joint damage. Neutrophils participate in autoimmune and chronic inflammatory processes partly through their ability to present antigens and stimulate T-cell responses.^[18]^ They produce matrix metalloproteinases (MMPs), elastase, and collagenase, which cause cartilage damage and bone destruction.^[19]^ Neutrophils also produce reactive oxygen species, which activate MMPs, leading to cartilage degradation and synovial inflammation.^[20-22]^ Elevated NLR may therefore be indicative of heightened neutrophil-mediated inflammation associated with RA development and disease progression.

Low serum albumin, indicated by higher NPAR, is correlated with systemic inflammation, liver dysfunction, and immune imbalance, confirming the role of albumin as a negative acute‑phase reactant. In addition, hypoalbuminemia may be associated with an increased risk of RA and is indicative of disease activity.^[23,24]^

Elevated NPAR has been reported to correspond to a greater risk of mortality in the general population as well as in multiple inflammatory conditions.^[24,25]^ A study involving 36,428 participants showed that NPAR was significantly positively associated with the risk of all-cause and cardiovascular mortality.^[26]^ NPAR is a predictor of mortality in patients with chronic kidney disease and is positively correlated with systemic inflammation and nutritional status.^[11,27]^ NPAR and other blood-based laboratory indicators of inflammation are positively associated with the risk of all-cause and respiratory disease mortality in adults and thus can serve as biomarkers for asthma.^[28]^ NPAR can accurately predict the occurrence of CVDs and is therefore a potential biomarker for early screening and prevention.^[29]^ NPAR is independently associated with 30-day all-cause mortality following ischemic stroke.^[30]^

In our cohort, NPAR was independently associated with higher all-cause and cardiovascular mortality, consistent with previous studies.^[31-33]^ Given that RA confers increased cardiovascular risk through chronic systemic inflammation,^[34]^ an inflammation-based index such as NPAR is biologically plausible as a prognostic marker. NPAR is calculated from 2 routinely available tests – a complete blood count (for neutrophil percentage) and serum albumin – rather than high-sensitivity assays,^[35]^ supporting its feasibility in resource-limited settings.

The major advantages of this study lie in its nationally representative cohort, long follow-up period, and rigorous confounder adjustment, all of which enhance the stability of the observed findings. We also applied survey weights and multiple imputation to address design complexity and missing data. Nonetheless, the study has limitations. First, the observational design cannot establish causality. Second, although adjustments were made for multiple covariates, residual confounding could not be entirely ruled out. Some factors not available in NHANES, including additional comorbid conditions, smoking burden, antirheumatic therapy, and RA-related disease characteristics, may have influenced the observed findings. Third, the application of listwise deletion may have decreased the effective sample size and introduced potential selection or estimation bias. Fourth, NPAR was assessed only at baseline and therefore could not capture longitudinal fluctuations, interindividual differences, or treatment-related changes over time. Serial measurements may provide a more reliable assessment of inflammatory status. Fifth, the present analysis was limited to US adults, which may affect the applicability of the findings to other populations. Additional studies involving diverse populations are needed to further confirm these results.

## 5. Conclusion

The NPAR is a biomarker of systemic inflammation and immune activation. NPAR independently predicted the risk of all-cause and cardiovascular mortality in a large nationally representative cohort of US adults with RA. These findings suggest that NPAR may serve as a practical biomarker for identifying patients with RA at elevated risk and could contribute to prognostic assessment in clinical settings.

## Author contributions

**Conceptualization:** Jun Dou, Ximei Wang.

**Data curation:** Beiqi Xu, Yanzhi Deng, Lilan Wang, Guang Yang, Dan Xie.

**Formal analysis:** Beiqi Xu.

**Funding acquisition:** Beiqi Xu.

**Investigation:** Yufan Wu.

**Methodology:** Ximei Wang.

**Project administration:** Dan Xie.

**Resources:** Dan Xie.

## References

[1] Babaahmadi M Tayebi B Gholipour NM. Rheumatoid arthritis: the old issue, the new therapeutic approach. Stem Cell Res Ther. 2023;14:268. 37741991 10.1186/s13287-023-03473-7 PMC10518102

[2] Black RJ Cross M Haile LM. Global, regional, and national burden of rheumatoid arthritis, 1990-2020, and projections to 2050: a systematic analysis of the Global Burden of Disease Study 2021. Lancet Rheumatol. 2023;5:E594–610. 37795020 10.1016/S2665-9913(23)00211-4 PMC10546867

[3] Cai W Tang XM Pang M. Prevalence of metabolic syndrome in patients with rheumatoid arthritis: an updated systematic review and meta-analysis. Front Med (Lausanne). 2022;9:855141. 35462993 10.3389/fmed.2022.855141 PMC9024100

[4] Kanagaraj P. Impact of self-management training on quality of life, medication adherence, and self-efficacy among rheumatoid arthritis patients. Int J Orthop Trauma Nurs. 2025;57:101178. 40186935 10.1016/j.ijotn.2025.101178

[5] Venetsanopoulou AI Alamanos Y Voulgari PV Drosos AA. Epidemiology of rheumatoid arthritis: genetic and environmental influences. Expert Rev Clin Immunol. 2022;18:923–31. 35904251 10.1080/1744666X.2022.2106970

[6] Liu B Wang J Li YY Li KP Zhang Q. The association between systemic immune-inflammation index and rheumatoid arthritis: evidence from NHANES 1999-2018. Arthritis Res Ther. 2023;25:34. 36871051 10.1186/s13075-023-03018-6 PMC9985219

[7] Sun XH Qian Y Cheng WQ. Characterizing the polygenic overlap and shared loci between rheumatoid arthritis and cardiovascular diseases. BMC Med. 2024;22:152. 38589871 10.1186/s12916-024-03376-1 PMC11003061

[8] Zhou EY Wu J Zhou X Yin YF. The neutrophil-lymphocyte ratio predicts all-cause and cardiovascular mortality among U.S. adults with rheumatoid arthritis: results from NHANES 1999-2020. Front Immunol. 2023;14:1309835. 38045692 10.3389/fimmu.2023.1309835 PMC10690944

[9] Choe JY Lee CU Kim SK. Association between novel hematological indices and measures of disease activity in patients with rheumatoid arthritis. Medicina (Kaunas). 2023;59:117. 36676741 10.3390/medicina59010117 PMC9862645

[10] Li YQ Wei WP Liu K. Neutrophil percentage to albumin ratio predicts all-cause and cardiovascular mortality in patients with diabetes or prediabetes from NHANES 1999–2018. Sci Rep. 2025;15:22863. 40594287 10.1038/s41598-025-06313-1 PMC12218579

[11] Liu M Gou YP Yang P. Neutrophil percentage-to-albumin ratio is associated with all cause and cardiovascular disease mortality in chronic kidney disease based on NHANES 2001-2018. Sci Rep. 2025;15:26546. 40696000 10.1038/s41598-025-12272-4 PMC12283933

[12] Song BW Kim AR Kim YK. Diagnostic value of neutrophil-to-lymphocyte, platelet-to-lymphocyte, and monocyte-to-lymphocyte ratios for the assessment of rheumatoid arthritis in patients with undifferentiated inflammatory arthritis. Diagnostics (Basel). 2022;12:1702. 35885606 10.3390/diagnostics12071702 PMC9317908

[13] Lee HN Kim YK Kim GT. Neutrophil-to-lymphocyte and platelet-to-lymphocyte ratio as predictors of 12-week treatment response and drug persistence of anti-tumor necrosis factor-α agents in patients with rheumatoid arthritis: a retrospective chart review analysis. Rheumatol Int. 2019;39:859–68. 30874873 10.1007/s00296-019-04276-x

[14] Zinellu A Mangoni AA. Neutrophil-to-lymphocyte and platelet-to-lymphocyte ratio and disease activity in rheumatoid arthritis: a systematic review and meta-analysis. Eur J Clin Invest. 2023;53:e13877. 36121342 10.1111/eci.13877

[15] Soeters PB Wolfe RR Shenkin A. Hypoalbuminemia: pathogenesis and clinical significance. JPEN J Parenter Enteral Nutr. 2019;43:181–93. 30288759 10.1002/jpen.1451 PMC7379941

[16] Yin FZ Hong HM Wang YQ. Mechanistic insights into NETs-induced osteogenesis inhibition in BMSCs of rheumatoid arthritis. Bone. 2025;198:117533. 40414474 10.1016/j.bone.2025.117533

[17] Edilova MI Akram A Abdul-Sater AA. Innate immunity drives pathogenesis of rheumatoid arthritis. Biomed J. 2021;44:172–82. 32798211 10.1016/j.bj.2020.06.010 PMC8178572

[18] Zhang L Yuan Y Xu Q Jiang Z Chu CQ. Contribution of neutrophils in the pathogenesis of rheumatoid arthritis. J Biomed Res. 2019;34:86–93. 32305962 10.7555/JBR.33.20190075 PMC7183296

[19] Carmona-Rivera C Carlucci PM Goel RR. Neutrophil extracellular traps mediate articular cartilage damage and enhance cartilage component immunogenicity in rheumatoid arthritis. JCI Insight. 2020;5:e139388. 32484790 10.1172/jci.insight.139388 PMC7406272

[20] Bolduc JA Collins JA Loeser RF. Reactive oxygen species, aging and articular cartilage homeostasis. Free Radic Biol Med. 2019;132:73–82. 30176344 10.1016/j.freeradbiomed.2018.08.038 PMC6342625

[21] Henrotin YE Bruckner P Pujol JPL. The role of reactive oxygen species in homeostasis and degradation of cartilage. Osteoarthritis Cartilage. 2003;11:747–55. 13129694 10.1016/s1063-4584(03)00150-x

[22] Wang X Fan DP Cao XX. The role of reactive oxygen species in the rheumatoid arthritis-associated synovial microenvironment. Antioxidants (Basel). 2022;11:1153. 35740050 10.3390/antiox11061153 PMC9220354

[23] Liu K Zhang L Zhao HM. Relationship between albumin and rheumatoid arthritis: Evidence from NHANES and Mendelian randomization. Medicine (Baltim). 2024;103:e39776. 10.1097/MD.0000000000039776 PMC11479416 39465845

[24] Wang D Su R Li RN Li XF Zhao H. Association of neutrophil percentage-to-albumin ratio with all-cause and respiratory disease-related mortality in US adults with asthma. BMC Pulm Med. 2025;25:313. 40611135 10.1186/s12890-025-03786-8 PMC12224837

[25] Liang H Pan K Wang J Lin J. Association between neutrophil percentage-to-albumin ratio and breast cancer in adult women in the US: findings from the NHANES. Front Nutr. 2025;12:1533636. 40357031 10.3389/fnut.2025.1533636 PMC12066505

[26] Han DZ Wu LL Zhou HB. Neutrophil percentage-to-albumin ratio and the risk of all-cause and cardiovascular mortality: a 20-year follow-up cohort study of 36,428 US adults. BMC Public Health. 2025;25:1483. 40264041 10.1186/s12889-025-22764-7 PMC12013024

[27] Rao JL Li YQ Zhang XH. The prognostic value of the neutrophil-percentage-to-albumin ratio for all-cause and cardiovascular mortality in chronic kidney disease stages G3a to G5: insights from NHANES 2003–2018. Ren Fail. 2025;47:2495861. 40336184 10.1080/0886022X.2025.2495861 PMC12064118

[28] Bi LG Liang JG Hu K. Neutrophil percentage-to-albumin ratio (NPAR) as a biomarker for asthma: a cross-sectional analysis of NHANES data. BMC Pulm Med. 2025;25:269. 40442659 10.1186/s12890-025-03701-1 PMC12121271

[29] Akgümüş A Duygu A Balun A. The relationship between neutrophil percentage-to-albumin ratio and infarct related artery patency in patients with acute coronary syndrome. J Health Sci Med. 2025;8:333–7.

[30] Lu YX Mao BJ Wang M Wan S. Neutrophil percentage-to-albumin ratio as a prognostic marker for mortality in Ischemic stroke patients. Int J Med Sci. 2025;22:2663–75. 40520906 10.7150/ijms.108493 PMC12163424

[31] Bai JY Lv TH Yu HM. The combined impact of neutrophil-percentage-to-albumin ratio and depressive symptoms on mortality in US arthritis patients: insights from NHANES (2005–2018). Front Public Health. 2025;13:1545250. 40115342 10.3389/fpubh.2025.1545250 PMC11922730

[32] Ding WQ La R Wang SH. Associations between neutrophil percentage to albumin ratio and rheumatoid arthritis versus osteoarthritis: a comprehensive analysis utilizing the NHANES database. Front Immunol. 2025;16:1436311. 39917306 10.3389/fimmu.2025.1436311 PMC11799277

[33] Zhang KJ Ma XD Zhou XC Qiu G Zhang CJ. Machine learning based association between inflammation indicators (NLR, PLR, NPAR, SII, SIRI, and AISI) and all-cause mortality in arthritis patients with hypertension: NHANES 1999–2018. Front Public Health. 2025;13:1559603. 40255373 10.3389/fpubh.2025.1559603 PMC12007114

[34] Huang XF Zhang YQ Hao WT Wu X Yang P. The association between neutrophil percentage-to-albumin ratio and cardiovascular disease: evidence from a cross-sectional study. Front Cardiovasc Med. 2025;12:1557507. 40438231 10.3389/fcvm.2025.1557507 PMC12116393

[35] Ganeshalingam S Wilson NJ Ciciriello S Antonipillai J Achuthan AA. Cellular interactions in maintaining an inflammatory microenvironment in rheumatoid arthritis. Mol Immunol. 2025;184:112–22. 40561673 10.1016/j.molimm.2025.06.008

